# Post Covid‐19 Condition as a Diagnosis: A Qualitative Study on Epistemological Tensions Among Experts in Sweden

**DOI:** 10.1111/hex.70463

**Published:** 2025-10-07

**Authors:** Anna Bredström, Sofia Morberg Jämterud

**Affiliations:** ^1^ Department of Culture and Society, Division of Migration, Ethnicity and Society Linköping University Norrköping Sweden; ^2^ Department of Thematic Studies, Technology and Social Change Linköping University Linköping Sweden

**Keywords:** diagnosis, epistemology, ethnicity, Post COVID‐19 condition, qualitative study, social categories

## Abstract

**Background:**

At the onset of the Covid‐19 pandemic, it became clear that some individuals experienced lingering symptoms after the infection. This condition, known as post Covid‐19 condition (or long Covid), is defined by WHO as persistent or new symptoms 3 months after the initial infection, lasting for at least 2 months, and not attributable to another diagnosis. Hence, the definition is very broad.

**Aim:**

This study aims to examine how post Covid‐19 condition as a diagnosis is viewed and interpreted by Swedish stakeholders, showing how these understandings carry a range of epistemological tensions. The study also seeks to understand the implications of these epistemological tensions for treatment and care organisation.

**Methods:**

Qualitative interviews with 36 experts and key individuals in Sweden have been conducted.

**Result:**

Experts agree that post Covid‐19 condition is a complex syndrome and that persons who suffer are in need of care. However, several tensions in post Covid‐19 condition as a diagnosis can be discerned. Most experts agreed on the gender and racial disparity where white women with Swedish background were a majority of post Covid‐19 sufferers, while migrant patients and the elderly are largely absent. In relation to social categories, the question if children can have post Covid‐19 condition is here a highly contested question. There is also disagreement on the aetiology of post Covid‐19 condition, with some experts viewing it as a new, unique condition requiring specialised treatment, while others see it as similar to other post‐viral conditions, treatable in primary care.

**Conclusion:**

The article concludes that experts are divided in their understanding and that this affects Swedish policy on post Covid‐19 care and treatment, showing that post Covid‐19 condition is not only a medical issue but also a political battleground where science, expert opinion and patient experience shape policy.

**Patient and Public Contribution:**

The article focuses on studying stakeholders' perspectives as these are key for informing public opinion and policy. In this article, all main organisations and authorities involved in post Covid‐19 care are represented. We also see patients as crucial stakeholders and representatives of patient organisations, as well as representatives of some migrant communities, who have been interviewed. The latter were included to gain insights from groups that were largely absent in post Covid‐19 care.

## 1 Introduction

Capturing a medical condition through a diagnosis can, in many respects, be seen as a medical advancement. For example, global classification systems like the American Psychiatric Association's *DSM‐5* are often considered valuable for medical research; they enable international comparative studies and create the conditions needed for evidence‐based targeted prevention efforts and successful treatments. However, diagnoses are complex in multiple ways. While medical science and health policy strive for clarity and well‐defined diagnoses, the reality for patients and healthcare is marked by comorbidity and complexity. This relates to some diagnoses more than others. Post Covid‐19 condition (hereafter post Covid) is one such diagnosis. Although several organisations, such as NICE [1], have proposed similar diagnostic criteria for post Covid, it is defined by the World Health Organization [2] as persistent or new symptoms occurring 3 months after the initial infection, lasting for at least 2 months, and not attributable to another diagnosis. In terms of symptoms, post Covid is highly heterogeneous. Most patients experience post Covid through fatigue, shortness of breath, and cognitive dysfunction [3], but there are reports of more than 200 other symptoms affecting patients' daily functioning [4]. Post Covid is also a patient‐made diagnosis, meaning that patients, through social media, raised awareness about their symptoms and need for care [5]. The cause of post Covid is also not established, which complicates the development of effective treatment options [6]. Nevertheless, post Covid remains a societal problem affecting a large number of individuals. The social science literature on diagnoses often highlights the importance of diagnosis for how diseases are dealt with by society at large, as well as experienced on the individual level [7]. Diagnoses are tools for classifying and serve an important administrative function in the allocation of resources and in the organisation of care. Diagnoses are also social constructs that pinpoint certain perspectives on the disease while making other aspects invisible [8]. They carry different connotations; some diagnoses are associated with stigma, while others appear more neutral [9]. Theories on medicalisation emphasise how social ‘problems′ have increasingly been given medical explanations, hence medical interventions are suggested as solutions [10]. Some diagnoses are also linked to various social categories such as gender, ethnicity, age and class in ways that may generate stigma and exclusion [9]. For example, research has shown that both gender and race affect psychiatric diagnoses such as depression and schizophrenia [11], and HIV/AIDS in its early days was so closely associated with men who have sex with men that authorities around the world struggled to conceptualise women as at risk [12]. The accelerating biotechnological development of recent decades has added another level of medicalisation, where genetics and neuroscience offer models for both ill health and social problems [13, 14].

This article takes its starting point in this ‘Sociology of diagnosis’ [15] and examines how post Covid as a diagnosis is viewed and interpreted by different Swedish stakeholders, and we show how these understandings carry a range of epistemological ‘tensions’. Some of these tensions can be traced to the interests of different professions and disciplines, others to the organisation of the Swedish healthcare system, and still others to social structures and broader societal discourses and social categories. In our analysis, we regard the diagnosis as a ‘boundary object’ [16], a perspective common in critical science studies, to capture ‘objects which are both plastic enough to adapt to local needs and the constraints of the several parties employing them, yet robust enough to maintain a common identity across sites’ [16, p. 393]. Regarding post Covid as a boundary object allows us to analyse the embedded tensions among different expert understandings and to trace the effects of different epistemologies in terms of treatment and organisation of care. We will also discuss the implications of our sociological approach for understanding the biopolitics around the development of post Covid health policy in Sweden.

### 1.1 Background

At the onset of the Covid‐19 pandemic, it became clear that some individuals experienced lingering symptoms after the infection, and patients' experiences became crucial in raising awareness of post Covid [17, 18]. This situation also emerged in Sweden, and in May 2020, the National Board of Health and Welfare published its first guidelines [19]. By the summer of 2020, reports in Swedish media highlighted that many were suffering from persistent symptoms even among those who had not required hospital care for the initial Covid‐19 infection [20]. The Swedish COVID Association was formed in July 2020 and became an important actor in advocating for help to those who were experiencing lingering symptoms. Initially, in the Swedish context, the phenomenon was first called ‘long‐term COVID’ (‘långtidscovid’), inspired by the Anglo‐Saxon term long Covid. However, soon after, the term ‘post COVID’ became common among patient representatives, authorities and healthcare providers, and Swedish policy documents align with the World Health Organization's definition [2].

In Sweden, the increasing number of affected individuals and the complex symptomatology led many regions to open specialised post Covid clinics with multiprofessional teams and in 2022, 12 out of 24 regions had such clinics. Currently, most of them have been closed, due to both a lack of patient base [21] and the recommendation that patients should be managed by primary care [22]. In Sweden, healthcare is decentralised and responsibility lies with the regional councils and municipalities. Primary care forms the foundation of the healthcare system and is responsible for providing essential health services. Some reports have identified deficiencies in the care of persons with post Covid [23], yet others have called for further examinations on the long‐term needs for children after Covid‐19 [24]. The ongoing and emerging symptoms reported by many patients, and the need for more knowledge, prompted the Swedish government (Ministry of Health and Social Affairs) in 2023 to commission several authorities to examine the state of knowledge of post Covid condition and provide knowledge of the diagnosis, care and rehabilitation treatment [25], including evaluating evidence on efficient research [26]. In August 2024, the National Board of Health and Welfare published one of the reports from this investigation, which included, in addition to post Covid, a number of other presumably post‐infectious conditions, including chronic fatigue syndrome (ME/CFS). The report underlines that the current state of knowledge remains unclear and highlights the importance of symptom relief and rehabilitation [22]. We will return to the report and the current situation in our concluding discussion, after presenting our empirical materials and our results.

## 2 Methodology

The empirical material that is analysed in this article consists of transcribed interviews with stakeholders, including representatives of all main organisations in Sweden involved in the development of post Covid diagnosis and treatment and other experts engaged in post Covid.

The interviewees were identified through either the organisation's webpage or through snowballing. Participants were strategically selected with regard to different experiences and professional backgrounds to gain as broad a picture as possible of the emergence of post Covid as a diagnosis in a Swedish context. In addition to policymakers at national authorities, we have interviewed medical researchers, different categories of healthcare personnel (e.g., doctors, nurses and physiotherapists), both at specialist clinics and in primary care, and representatives of post Covid clinics. We also consider patients as important stakeholders, and we have interviewed representatives of patient organisations as well as representatives of some migrant communities (based on language: Arabic, Dari, Persian and Somali), the latter were included to gain some insights from a group of patients that were largely absent in post Covid care.

In total, we conducted 16 individual interviews and 6 group interviews with 2–6 participants in each group. The groups were composed of single stakeholders, and there were no overlaps between individual and group interviews. For ethical reasons, we have chosen only to use individual quotes, even though these can be from group interviews.

A total of 36 interviewees participated in the study; approximately 45 people were approached and those that declined did so mainly due to time constraints and other obligations. The sample included 29 women and 7 men, and the age range spanned from 30 to 70 with the majority being between 40 and 60. Most interviewees were based in Stockholm, reflecting the concentration of national authorities in the capital. However, the healthcare representatives included participants from regions across the country, ranging from the south to the north.

The interviews took place between November 2022 and September 2024 and were conducted by both authors together. The interviewers have extensive experience in conducting qualitative interviews and analysis. Their background in medical sociology, medical humanities and medical ethics informed the data collection and analytic process. The interviewees were informed by letter and orally about the project. Participation was voluntary and participants gave written, informed consent before the interview. Both individual and group interviews lasted between 1 and 2 h, and they followed the same thematic interview guide that covered the understanding of the post Covid diagnosis; views on treatment and care; and views on vulnerable groups. All interviews were recorded, transcribed verbatim and pseudonymised. In the following analysis, no names of individuals, places or institutions are mentioned. The study was guided by the Standard for Reporting Qualitative Research [27].

The interviews have been coded independently by both researchers according to standard principles for qualitative analysis [28] with a focus on how the diagnosis is understood by the informants. The coded material was divided into main categories and subcategories. The main categories were social categories and vulnerability to the disease, aetiology, treatment and symptom expression. The subcategories were gender, race, class and age as specific social categories affecting the understanding of post Covid epidemiology; post Covid as a new and unique disease or an expression of a well‐known virus infection sequela; and post‐Covid treatment as a primary care or specialist care illness. The analysis is presented in the “Results” section, divided into two broader themes that each cover the different subcategories.

## 3 Results

### 3.1 Social Categories and Post Covid

A recurrent theme in the interviews concerns the ways in which social categories of gender and race/ethnicity are articulated in relation to post Covid. Throughout the history of pandemics, there are countless examples of how risk exposure can lead to both stigma and prejudice about why some people get sick and others do not [29]. In the early days of the Covid‐19 pandemic, speculations were also spread that made people of Asian descent feel singled out as carriers [30]. In the Swedish context, a social epidemiological discourse was soon established, which suggested that vulnerability to risk followed from the structural conditions that made it difficult for some groups to protect themselves from the virus [31]. People who lived in crowded conditions, were in need of public transport or had jobs that could not be done from home were more often infected [32]. Together with the increased risk of severe illness among the elderly, other at‐risk groups such as people with cardiovascular disease, the pandemic thus hit certain social groups harder. In addition to the elderly, ‘non‐western migrants’ were considered particularly vulnerable. Already during the first months of the pandemic, there were reports of over‐representation of men with non‐European backgrounds among those in need of specialised care [33, 34].

When comparing Covid‐19 with post Covid, a diametrically different picture emerges, both in terms of gender and ethnicity. While more men than women were affected by severe disease in the case of Covid‐19, the majority of post Covid sufferers are women. And while Covid‐19 patients with a migrant background were over‐represented among those affected by severe disease, people racialised as ‘migrants’^1^ seems to be completely absent among post Covid patients. The only exception would be those who received their post Covid diagnosis due to long rehabilitation after ICU care. They are, however, absent at the specialist post Covid clinics and are not seen as a typical post Covid patient as their condition is seen as primarily linked to their ICU experience. This gendered and racialised epidemiological description is confirmed both by the limited statistics available [22, 36] and by those we interviewed. In the interviews with doctors who have met many patients at post Covid clinics, it was expressed in terms such as ‘there was 70% men in one group [severe Covid cases], yet more than 80% women in the other’ [post Covid patients]. One doctor highlighted the absence of migrant patients by pointing out that during these 4 years that he had worked at the post Covid clinic, he had not had a single patient that required a language interpreter, something very unusual in healthcare when your patients are a general sample of the population.

Some of the experts speculated on the reason for this conspicuous absence of migrant patients in the post Covid data. One expert suggested that it might be because many of them are unemployed and therefore do not need to be diagnosed to the same extent as those who are dependent on the National Health Insurance. Others pointed to varying care‐seeking behaviours among patient groups and different interpretations of symptoms, possibly due to stigma or cultural differences. Social background, particularly education, is also highlighted as a possible explanation for why some people are diagnosed and others are not:

I imagine that if you're a very high achiever, and you get the slightest bit of cognition deficiency or something, then you′re very observant of that, because you're so dependent on those functions, like in your job maybe, or and then you're kind of looking for help, while those who maybe don't kind of, what can I say, have those kind of like high performance jobs, like maybe not [look for help]…. They might notice that they are more tired than usual, that they might not have the same…. I don't really know, or don't have the energy, or don't know, or just don't go.

One of the interviewees questioned the possibility for the concerned group to reach specialist care given that it requires a lot of social and cultural capital. This interviewee thus focused on the structural and institutional constraints that hindered some groups of patients to access the care they needed.

In addition to ethnicity, class/education and gender, *age* also emerges in the interviews as a social category of importance. Age was often highlighted by the authorities during the pandemic, in that the elderly were pointed out as an at‐risk group for severe illness. Several interviewees pointed out that in relation to post Covid, the elderly are relatively invisible. Some experts were self‐critical and argued that the elderly might not be picked up by health services precisely because they are older; fatigue and weakness might be interpreted as symptoms of ageing rather than linked to a previous Covid infection, for example. In the case of children, however, experts had different views. Some emphasised that children were also a neglected group and said that there were children who were very badly affected by post Covid:

We expect a couple of percent of all children to have it [post Covid], in some way to some extent. Then there may be only a small percentage of them who have extreme problems. But 2%–5% of all children suffer from post Covid, which is comparable to a concussion. The majority of them have a spontaneous recovery anyway, within a few months or 6 months. But then there is a subpopulation that suffers for a longer time. That's the one you might need to support above all.

Others argued that although children in general were severely affected by the pandemic in terms of online schooling and cancelled leisure activities, these experts considered both severe Covid and post Covid to be a rare phenomenon among children; ‘the numbers were very small’, ‘there were very few children and young people [seeking specialist care]' one expert concluded.

The latter view that post Covid is not such a widespread problem for children is also the most common interpretation. However, in the public debate, the question of children has been raised as a blind spot amongst policymakers [37, 38, 39] and the fact that children are addressed (alongside the elderly, women and men) in the abovementioned governmental investigation [25] indicates that there was political influence among those who were concerned about the invisibility of severe disease in children.

### 3.2 Aetiology, Treatment and Organisation of Care

Moving on to the second theme focusing on how the experts understand the cause, and by extension, the treatment of post Covid, it is important to return to the heterogeneity of the disease. As mentioned earlier, post Covid not only encompasses a range of different symptoms, but is also defined in terms of *time*. For those who became seriously ill and required advanced care, it was not surprising that it took a long time to recover. Most of the experts do not explicitly or indirectly include people with post‐intensive care syndrome in their understanding of post Covid patients, even if they are formally diagnosed as such. Instead, the experts referred to people with long‐lasting (or new) symptoms who had not been as severely ill in the early stages. Some accentuated this perception that post Covid affects people who are otherwise healthy (as opposed to those who needed ICU care, many of whom were vulnerable to the infection due to their previous health status). A recurrent narrative is how they were struck by their first encounters with a post Covid patient:

Interviewer: But what was it that shocked you when you met them? You said you were almost shocked.

Informant: Yeah, […] [she] was like this super active person who works out at least three, four times a week, manage to have a full time job and stuff […] but when I did like this 6 min walk test, then just: ‘Is this for real, like?’

Interviewer: Because she was so ill?

Informant: Yeah, and […] I′m looking at the saturation meter, and the pulse was just wow, wow. Then you get like this: ‘Is there something wrong with the meter?’. And then it was also that, suddenly I just see how she drops in saturation, […] So that was probably what made me just: ‘oh shit, what is this?’.

In these interviewees, the novelty and uniqueness of post Covid was often emphasised. ‘We know very little about this new virus,′ they reasoned, and these experts believed that post Covid will continue to be an urgent health issue, even if Covid‐19 gradually turns into more of a common cold. Thus, more research and knowledge are needed, and post Covid needs to be treated by specialists. They were also very sceptical about transferring responsibility from the multidisciplinary post Covid clinics to primary care. Primary care staff, they argued, have less knowledge about this complex condition. Ignorant healthcare workers might even question the diagnosis itself, or treat it with suspicion, making it harder for post Covid patients to get the treatment they need. Both adequate knowledge and the right attitude were seen as crucial to the ability of the post Covid patient to get the help they need to get well. Focusing only on treating the symptoms, without a deeper understanding of post Covid, could actually have undesirable effects. A recurring example was when exercise was suggested as part of the treatment for symptoms of fatigue and depressed mood, symptoms that overlap with other diagnoses such as burn‐out and major depression. Not knowing the cause of the symptoms, or not having the correct diagnosis, could lead to a worsening of the symptoms rather than treatment:

…and that can be dangerous, because we sometimes see those who are affected that they can have the same symptoms but have completely different causes. […] It can even be the case that what is good for one patient can be harmful for another, for example, a patient suffering from psychological problems may feel good about physical exercise, while a person who has problems with heart rate regulation may have to avoid certain types of exercise immediately.

This discourse of separating post Covid from other complex illnesses with overlapping symptoms was also surrounded by theories of underlying biomedical causes. The idea that post Covid was a result of residual virus in the body was one such theory. While the common definition saw post Covid as something that happened *after* the initial infection had healed, both patient advocates and some prominent doctors challenged this narrative and saw post Covid as an ongoing disease process:

That has also been a misconception, we think. That people just talk about everything being a recovery from the acute infection. But we ask ourselves the question: If you have a fever for two and a half years, can you really still talk about a recovery then?

This narrative of post Covid as a unique diagnosis requiring specialised care is challenged in other interviews. For example, it is argued that there are many similarities with other post‐viral conditions and that having long‐lasting symptoms after a viral infection is not a new phenomenon.

But for me it is not a new disease. This has existed … post‐viral […] We see the same problems with MERS, SARS, influenza. This fatigue has also existed after other infectious diseases or other diseases. So I don't think it's particularly strange.

These experts question the idea that post Covid is associated with an ongoing infection. Rather, post Covid is seen as a secondary disease, a sequela, that occurs sometime after the primary infection has healed. The fact that post Covid is a sequela does not mean that there is no residual virus. One of the interviewees, with expertise in infectious diseases, points out that ‘with today's sensitive methods you can often find residues and it's actually the case that a fair proportion of our genetic material is actually virus residues’, but explains that there is no evidence that remaining symptoms can be treated by targeting the original infection. Similarly, these experts did not consider it as important as the experts above to distinguish between post Covid and diagnoses with similar symptoms. On the contrary, they saw similarities and overlaps as important information. For example, several felt that the over‐representation of well‐educated, middle‐aged women among post Covid patients may well be related to the fact that there are many who have an underlying vulnerability due to previous experience of burn‐out or depression, diagnoses where the same category of patient is also over‐represented:

And then we have later seen that one group is over‐represented, these women in their 30s and 40s, previous fatigue depression […] Yes, then you can imagine that what you see, which the patient describes and is quite clear about is that it is not fatigue. It's not depression. I've had that before. It doesn't feel the same. And then I think that for me it is still a common course of illness after Covid. Long drawn out, where you push yourself too hard. And then they end up in their exhaustion again. That's my explanation.

Finally, there is also less focus on cure in these interviews, instead emphasising adaptation and time, that is, that ‘most people get better eventually’. It was also felt to be ethically unacceptable to prioritise post Covid over other conditions, especially as the degree to which a post Covid patient is affected by their symptoms varies greatly. Therefore, in their view, and in contrast to the experts above, primary care would be the obvious place to treat post Covid, and it is also well placed to deal with comorbidities and complexity, they argued:

But somewhere in this we know very little about the disease and therefore it is also the case that the role of primary care is to hold the whole together somewhere. So it is clear that there are few people who do not have something else with them in this as well. And that's another reason why the entrance is still via primary care […] there is a general direction that the hub is still in primary care, but that some may need to be referred onwards.

This should not be interpreted as a denial of the suffering that post Covid can bring to an individual; on the contrary, almost all could speak of seriously ill patients whose lives were severely curtailed when they developed post Covid. Similarly, everyone agreed that more knowledge is needed about both the disease and the treatment, and perhaps there is sometimes a case for specialised care for the most seriously ill, but not for everyone.

## 4 Discussion

The expert interviews in our study confirmed that white/Swedish, often well‐educated, middle‐aged women were strongly over‐represented among those with the disease. This gendered (and racialised) epidemiology also serves as an important entry point to the main argument in this article, which queries the epistemological tensions between different experts.^2^ However, as mentioned above, our analysis sets out from the understanding that the diagnosis of post Covid works as a ‘boundary object′ that holds different, and sometimes contradictory, understandings of the illness together. In our analysis, we found a tension between those who see post Covid as something new, requiring specialist treatment and research‐based knowledge, and those who argue the similarity both to previous pandemics and to post‐viral infections in general. The latter tend to see rehabilitation and adaptation as a strategy rather than a cure and believe that symptoms are secondary and therefore separate from the original infection—as opposed to the argument that it is a continuation or fragments of the virus that cause new symptoms.

Zooming in then on how the experts understand *why* (white/Swedish, middle‐class) women are overrepresented highlights the importance of these divergent views. For those emphasising the similarities with other post‐viral infections, women are over‐represented in relation to post Covid *because* they tend to have a history of unexplained pain/ME, burn‐out, depression and anxiety, thus the epidemiological pattern does not come as a surprise. The other group of experts, by contrast, seek to avoid exactly that link; by referring to ME, burn‐out and depression and anxiety, they seem to think, post Covid risk losing its status and become one of those female‐dominated non‐specific disorders that have struggled to gain legitimacy and resources within health policy and care.

Hence, in the Swedish public debate, there are important voices arguing against conflating post Covid with other unexplained illnesses that women suffer from, and they have been active in lobbying for new governmental guidelines on post Covid. When the decision was later made to proceed with this [25], it was clear that it would address many core issues raised by these voices: the need for specialist care and a specific knowledge authority, and the need to investigate how children are affected by post Covid. When the investigation was launched, many expressed their support, such as patient organisations [42]. However, a year later, when the report was published [22], some expressed their disappointment with the guidelines. The final report emphasises similarities and links with other (presumably) post‐viral conditions, and a reliance on primary care and rehabilitation [22], which led to some questioning the conclusions, arguing that they were not in line with international policy or established evidence and also that some of the advice was directly incorrect for patients with post Covid [43, 44, 45].

For our analysis in this article, the opposing views and charged opinions are a good starting point for a sociological analysis of the diagnosis. It shows how, in our case, post Covid is not only a complex medical condition, but also a space for a biopolitical act, where scientific facts, expert opinions and patient experiences are mobilised in different ways to form alliances and new policy agendas. For complex diseases with no clear aetiological picture, understanding this interplay is of utmost importance, as both policy initiatives and research will inevitably be part of this tug‐of‐war.

### 4.1 Strengths and Limitations of the Study

The strengths of this study lie in the thorough coverage of the key institutions and experts that played a pivotal role in shaping the post Covid agenda in Sweden. Another strength is the inclusion of patient opinion from beyond recognised patient organisations, capturing groups that would otherwise be silent. In our case, this includes patients from migrant backgrounds. A limitation of the study is its single focus on Sweden; an international comparison would have shed light on the potential specificity of the Swedish case.

## 5 Conclusion

In this article, we demonstrate that experts do not agree on what post Covid means and that this affects Swedish policy on post Covid care and treatment. Our study also reveals that post Covid diagnosis is unevenly linked to different social categories, with well‐educated, middle‐class, Swedish‐born women being over‐represented among sufferers, whereas non‐Swedish migrants are generally absent. Age as a social category is also present in the expert discourse, with children being a contested issue. The key conflict among experts concerns the extent to which post Covid should be considered a unique illness requiring specialist care or whether it has more in common with other long‐term illnesses. In conclusion, we argue that post Covid should be viewed as a boundary object comprising different—and sometimes opposing—views. The effect of this is a topic for sociologists of diagnosis.

## Author Contributions

**Anna Bredström:** methodology, investigation, conceptualization, writing, formal analysis, review and editing (equal). **Sofia Morberg Jämterud:** methodology, investigation, conceptualization, writing, formal analysis, review and editing (equal).

## Ethics Statement

The project is approved by the Swedish Ethical Review Authority (Dnr 2022‐03899‐01).

## Conflicts of Interest

The authors declare no conflicts of interest.

## Data Availability

The data that support the findings of this study are available upon request from the corresponding author. The data are not publicly available due to privacy or ethical restrictions.

## Endnotes

1 In contemporary Sweden, discourses of race and ‘otherness’ are largely articulated in terms of ethnic and cultural difference rather than race per se, and the (non‐European) ‘migrant’ is the quintessential racialised subject [35].

2 Research indicates that, despite the fact that there is a dominating invisibility of migrants in the mainstream discourse, many groups of migrants are highly vulnerable to post Covid following the fact that they were very seriously affected by the virus itself [40]. We discuss post Covid as a racialised epidemiology in a separate article where key issues are migrants' lack of trust in Swedish healthcare and experience of institutional racism [41].
